# Towards reducing behavioral risk factors of non-communicable diseases among adolescents: protocol for a school-based health education program in Bangladesh

**DOI:** 10.1186/s12889-019-7229-8

**Published:** 2019-07-25

**Authors:** Marium Salwa, Md Atiqul Haque, Md Khalequzzaman, Mohammad Abdullah Al Mamun, Mahfuzur Rahman Bhuiyan, Sohel Reza Choudhury

**Affiliations:** 1 Chi Research & Infotec Ltd, Dhaka, Bangladesh; 2 Department of Public Health and Informatics, Bangabandhu Sheikh Mujib Medical University, Dhaka, Bangladesh; 3 Department of Epidemiology & Research, National Heart Foundation Hospital & Research Institute, Dhaka, Bangladesh

**Keywords:** Behavioral intervention, Health education, Adolescent, NCD risk factors, Bangladesh

## Abstract

**Background:**

Developing strategies aimed at reducing behavioral risk factors and hence the prevalence of non-communicable diseases (NCDs) is a major challenge to the policy makers today. Like the same age group worldwide, the prevalence of obesity, unhealthy dietary habit, physical inactivity, smoking and alcohol intake is high among the adolescents of Bangladesh. Studies showed promising results of an early intervention at adolescent age in reducing the likelihood of NCDs at adult age. So, this study is designed to implement a behavior change intervention and evaluate the effectiveness of the intervention in reducing the behavioral risk factors of NCDs among the adolescents of Bangladesh.

**Methods:**

A before-after designed intervention study will be conducted in two randomly selected secondary schools- one will be selected randomly as intervention school and the another as control school. A baseline survey will be conducted among the students of both schools by a pre-tested questionnaire to attain their current status of knowledge, attitude and practices related to NCDs. Afterward, students will be enrolled in the intervention group who will meet the eligibility criteria from the intervention school. The intervention will be given through a health promotion session to a group of students, not more than 25 at a time, by trained facilitators. A post-intervention end line survey will be conducted among all the participants from both schools using the same questionnaire 3 months after the baseline survey.

**Discussion:**

An intervention has been developed based on some principals of two psychosocial theory- Motivational Interview and Social Cognitive Theory. Emphasis will be given on motivating the adolescents towards a healthy lifestyle, supporting self-efficacy to be changed, guiding self-regulatory ways along with facilitating desired changing process by empowering them with choices about the preventive measures of NCDs. This intervention is expected to increase awareness by equipping the adolescents with specific knowledge and skills and thus, facilitate an eventual change in their practiced risk behaviors. Besides, this intervention will address multiple behaviors at a time, and will be delivered to a group of adolescents, to attain the cost-effectiveness and thereby making it more realistic in the resource-poor context of Bangladesh.

**Trial registration:**

ClinicalTrials.gov NCT03975335, registered on 01.06.2019. Retrospectively registered.

**Electronic supplementary material:**

The online version of this article (10.1186/s12889-019-7229-8) contains supplementary material, which is available to authorized users.

## Background

Towards addressing the non-communicable diseases (NCDs) epidemic in Bangladesh, effective measures are needed to stifle its emergence. In the case of NCDs, it is useful to start the prevention process at the adolescent stage [1] since most of the behavior related risk factors of NCDs begin to demonstrate at this age [2]. Targeting adolescents also has the additional benefit of reducing the life-time cost of healthcare by addressing the behaviors at the outset [3, 4].

Adolescents of Bangladesh, like the rest of the world, are exposed to significant amount of NCDs risk factors such as obesity [5], less fruit and vegetable intake [6], physical inactivity [7], smoking [8] etc. Given modifiable risk factors are prevalent in large numbers, intervention that aims to change behavior can be an effective method of halting the growth of NCDs. Several theories on how human behavior is shaped have been discussed and evaluated over the past decades or so. For example, attitude towards a behavior and subjective norm or social influence have been emphasized to influence human behavior in many theories including theory of planned behavior [9]. Meta-analysis supported the theory that there is a strong relationship between attitude and behavior, and behavioral intention plays a major role as a mediator between them [10]. Furthermore, greater attitudinal consistency is observed amongst the better informed [11]. Self-efficacy expectation or personal belief to accomplish a change in behavior described by Bandura in his Social Cognitive Theory (SCT) [12, 13] was proven to have successfully predicted behavioral intention [14, 15]. Moreover, adolescents were found in need of tremendous psychological support or motivation to shape positive behaviors [16]. Motivational Interview (MI) postulated by Rollnick & Miller (1995) [17] showed promising results to this effect.

Understanding behavioral risk and protective factors can positively influence the development of intervention programs [18]. Theory-based intervention has proven to be an effective method in driving behavioral change. However, choosing the right intervention technique is a difficult task and needs to be experimented and evaluated in context specific settings [19, 20]. Studies suggested that the choice of a suitable theory base for an intervention should begin with identifying the problem, goal, and units of practice [18]. Also, intervention targeting multiple behavior has been proven to have greater impact than single behavior intervention [21, 22].

Considering all these aspects, an intervention has been designed to reduce NCDs related risk behaviors such as unhealthy dietary habit, physical activity and smoking among the adolescent of Bangladesh considering some of the components of MI and SCT. In this intervention, emphasis is given on evoking adolescents’ internal desire to change, supporting self-efficacy to be changed, guiding self-regulatory ways along with facilitating desired changing process by empowering them with choices and skills. It is to clarify that, this study will not measure the efficacy of the theory of MI or SCT, rather the effectiveness of the proposed intervention will be evaluated which has been constructed based on some principles of the former theories. This intervention is expected to increase awareness by equipping the adolescents with specific knowledge, motivating them, and facilitating an eventual change in their practiced risk behaviors.

## Objectives

### General objective

To design and investigate the efficacy of a health education intervention to reduce NCD related behavioral risk factors among adolescents in institutional settings of Bangladesh.

### Specific objectives

1. To conduct a baseline survey to evaluate the knowledge, attitude and practices about NCD risk behaviours such as unhealthy diet, physical inactivity, smoking etc. among the respondents.
2. To develop a health education intervention based on the baseline survey to reduce NCDs related behavioural risk factors among adolescents.
3. To apply the health education intervention to the target group through effective communication approach and tools.
4. To conduct a post interventional survey with the same questionnaire used in baseline data collection.
5. To evaluate the intervention by comparing pre and post intervention data.

## Methodology

### Study design and setting

A before-after designed intervention study will be conducted in two randomly selected secondary schools in a semi-urban area of Dhaka, capital of Bangladesh. One school will be selected randomly as intervention school and the other one as control school. Possibility of diffusion of intervention contents among students from one school to another will be minimized by selecting the schools at a reasonable distance.

### Participant recruitment and data collection

The study will follow a fixed study design (Additional file 1). Sample size is determined based on a previous study where a mean difference of BMI was found 1.48 between intervention and non-intervention adolescent groups after 3-months of motivational interview [23]. Expecting a similar difference, calculated sample size is 274 in each group considering effect size (E) = 1.48; standard deviation (S) = 5.98; standardized effect size (E/S) = .25; significance level, α (two-sided) = 0.05; β = 1- power = 0.2, non-response rate = 5%]. A complete list of grade-ten students will be prepared after collecting their class roll and batch number from the administrative offices of the selected schools. All the students of grade-ten will be invited to participate in the study through announcement and personal contact prior to the date of data collection.

At first, a baseline survey will be conducted among the grade ten students of both schools by a pre-tested questionnaire to attain their current status of knowledge, attitude and practices related to NCDs. Then, students from the intervention school who reported at least two NCDs risk behaviors will be invited to participate in a health education session through phone call to their parents. Selected students who will present at the day of intervention will be considered having implied consent from their parents and will be enrolled in the intervention group up to the attainment of the required sample size. Enrolled students will be given intervention through health education session. Students with any kind of physical disability or limited movement will be excluded from the study. Students from the control group will receive a one-hour session on carrier guidance. Students will be kept blind about the intervention they received. After 3 months of the baseline survey, a post-intervention survey will be conducted among the participants from both intervention and control group using the same questionnaire.

### Data collection tool

A self-administered questionnaire is being constructed after reviewing several survey questionnaires in this field including Global School Health surveys [24], STEPs Survey of NCD risk factors [25], a Mongolian survey questionnaire [26] etc. The questionnaire is being constructed first in English, then translated into Bangla and again back translated into English by experts to ensure the translation. Then the questionnaire will be pre-tested among a similar population and modification of the questionnaire will be done accordingly. Finalized questionnaire (Additional file 2) will be used both in pre-intervention baseline and post-intervention end line survey.

There will be four sections in the questionnaire- the first section of the questionnaire will contain questions on socio-demographic information of the participants. The second section will contain questions about knowledge, attitudes and practice related to NCDs e.g. cardiovascular diseases, stroke, diabetes, hypertension, cervical cancer, etc. Third section will cover questions on knowledge, attitudes, perceived barriers and practice related to behavioral risk factors of NCDs e.g. diet, physical activity, smoking and alcohol, and the fourth section will hold physical measurement of the respondents such as height, weight, blood pressure, pulse, waist and hip circumference etc.

### Outcome variables

Following outcome variables will be measured.

### Knowledge

Knowledge about NCDs and risk behaviors will be measured by 25 knowledge measuring questions scoring 54 in total. Answer to the questions will be regarded as correct or incorrect based on information from standard medical textbooks and guidelines.

### Attitude

Attitude towards NCDs and its risk factors will be measured by nine attitude measuring questions in a Likert Scale.

### Practice

Practice questions will be used to evaluate dietary habit, physical activity, smoking, alcohol and substance abuse.

Based on WHO recommendations and standard practices for adolescent age, the following practices will be measured in this study.

1. Dietary habit: presence of at least two of the following four habits will be accounted as having dietary risk behavior
  i. *Inadequate fruit consumption:* Less than five servings of fruits per day
  ii. *Inadequate vegetable consumption:* Less than five servings of vegetables per day
  iii. *Excessive salt consumption:* Taking extra or raw salt during every meal
  iv. *Sugar Sweetened Beverage (SSB) consumption:* Consuming SSB more than 3 days per week
2. Physical activity*:* Not meeting 60 min physical activity of moderate intensity per day will be regarded as physically inactive.
3. Smoking and alcohol: Smoking regularly in the last 30 days or exposing to passive smoking more than 3 days per week will be accounted as in risk of smoking. Any amount of alcohol intake or any substance abuse in the last 30 days will be regarded as at risk.

### Physical measurement

#### Blood pressure

Blood pressure will be measured by an automatic digital sphygmomanometer (HEM-8712, Omron, Kyoto, Japan). Students will be asked to sit down on a chair and rest their right elbow on a table. Blood pressure will be measured three times allowing at least 5 min interval between each measurement. The mean of second and third measurement will be taken into analysis. Presence of systolic blood pressure ≥ 130 mm of Hg and/or diastolic blood pressure ≥ 80 mm of Hg will be considered as having high blood pressure.

#### Height

Height will be measured by a portable and non-stretchable measuring tape. Students will be asked to remove their shoes and head gear, if any and stand straight on the flat floor against a wall, facing the interviewer. Their heels will be in touch of the wall. Interviewers will make sure that their eyes will be at the same level as the ear. Height will be recorded in centimeter to the nearest millimeter.

#### Weight

Weight will be measured by portable digital weighing machine (Medisana Bathroom Glass Scales). The machine will be placed on a flat floor. Students will be asked to remove their shoes and extra cloths, if any, and stand straight on the machine hanging their arms by their sides. Weight will be recorded in kg.

#### Body mass index (BMI)

The ratio of body weight in kilogram and the height in meter square will be considered as BMI. Classification of BMI will be done as following-.

Underweight (Less than 18.5); Normal weight (18.5–24.9); Overweight (25.0–29.9) and Obese (More than 30.0).

#### Waist and hip circumference

Waist and hip circumference will be measured by a stretch-resistant measuring tape and recorded in centimeter to the nearest millimeter. Waist circumference will be measured at the midpoint between the lower margin of the last rib and the iliac crest and hip circumference will be measured at the widest part of the buttock with the tape parallel to the floor following WHO guideline [27]. For both the measurements, students will be asked to stand straight with feet close together and arms at the side. The measurement will be taken at the end of normal expiration.

### Data management plan

Collected data will be entered into the pre-designed SPSS file (Version 21) and then checked, cleaned and recoded where needed. Only the authorized personnel will be able to access data to avoid any potential manipulation. Data set will be archived and only available on request once the study is over.

### Statistical analysis plan

Descriptive analysis: Socio-demographic data of the participants e.g. gender, parental occupation, parental education status, family history of NCDs, family living standard will be obtained for both intervention and control school. All categorical variables will be summarized in frequency and percentages and continuous variables in mean and standard deviation. To estimate the association of these characteristics of intervention and control group, t-tests and chi-square tests will be done.

Inferential analysis: To estimate the efficacy of the intervention, before-after data will be analyzed and paired t-test and McNemar’s chi-square tests will be done in this regard. Also, comparison will be done between the intervention and control group for pre and post-intervention status of knowledge, attitude and practices.

Association analysis: Binary regression analysis will be done to estimate the association between socio-demographic variables with changes in the outcome variables- knowledge, attitude, and practice for both groups. Also, to estimate the association of knowledge score and attitude with the change in self-reported practices, regression analysis will be done.

### Quality control measurement

Quality of the data and adherence with the protocol will be ensured by a quality control panel consisting of four senior physicians from Bangabandhu Sheikh Mujib Medical University (BSMMU).

The facilitators to deliver the intervention will be practicing physician who will have experiences on individual patient counseling. Selected facilitators will be trained on the intervention by written dialogues addressing the sequential framework of the intervention model in a 5-days workshop. Then they will have to perform dry run. Their lectures will be recorded and then scrutinized to ensure standard and similarity between the lectures. A supervisor team consisting of a psychologist, a child health specialist, a public health specialist and a nutritionist will be present during the dry run and at the actual intervention session to ensure the quality. All the sessions will be audio-recorded to measure the quality of the intervention and to confirm its reproducibility. A SPIRIT checklist has been filled up for this protocol to ensure its standard (Additional file 3).

### Benefits and risks of participation

Every participant will receive a stationery bag as gift containing a notebook, a pencil, an eraser and a sharpener, costing about $ 2, upon completing the baseline survey. No monetary incentive will be provided.

Any information about the risk behaviors of the individual participants will not be disclosed. Intervention will be given in groups of students where participants possessing all kind of risks will take part instead of separating them in specific group such as physically inactive group or smoker group etc. Thus, we will try to minimize any perceived physical or psycho-social risk for participating in this study. No invasive procedures will be involved.

### Ethical consideration

This study protocol got ethical clearance from Institutional Review Board (IRB) of BSMMU (BSMMU/2018/5958). Written permission will be obtained from the authorities of both schools and informed written assent will be collected from every participant prior to participation. Parents or legal guardians of the selected students will be informed about the intervention over telephone and through notice on school diary. If the parents send their children on the day of intervention, it will be regarded as their implied consent [28] to let their children participate in the health education intervention. No written consent will be obtained from the guardians other than the implied consent which has been approved by the IRB, BSMMU.

All the physical measurement will be taken in a separate room maintaining privacy and confidentiality. Female volunteer medical students will measure blood pressure and anthropometric measurements of the female participants.

### Reporting of the result

It is decided to disseminate this intervention and the findings of the proposed study through a dissemination seminar and through reporting on it. The Transparent Reporting of Evaluations with Non-randomized Designs (TREND) statement checklist will be followed while reporting this study to ensure maintaining highest standard and getting maximum possible benefit out of this study in creating evidences.

### The intervention

Potential candidates for intervention will be divided into several groups based on their roll numbers. Each group will contain no more than 25 students. Then the intervention will be delivered to each group through health promotion sessions.

The intervention has been developed with the help of a psychologist, a nutritionist, a child health specialist and some public health specialists having experiences to work with adolescent health. Content of the intervention will be further improved and customized after baseline survey addressing the barriers to healthy behaviors among the target population.

The conceptual framework of the expected change through this intervention is as follows:

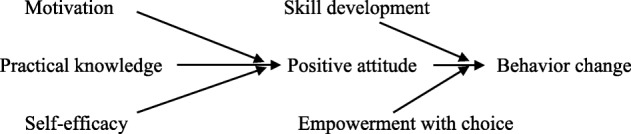



Intervention will be given on the following issues:

Issue 1: Diet

1. Understanding that there is an effect of every food we eat on our body and health besides alleviating our appetite
2. Demonstrating all the foods we eat every day and grouping them according to their nutritional values
3. Understanding the metabolism and function of different food groups- carbohydrate, protein, fruits and vegetables, fries and fast food, soft and energy drinks
4. Understanding and identifying healthy and unhealthy food options depending on its function
5. Understanding and identifying the link between unhealthy food options and NCDs
6. Raising curiosity on what should be the food options to prevent NCDs

Issue 2: Physical activity

1. Demonstrating the concept of physical activity and sedentary behavior
2. Understanding and identifying the link between physical inactivity and NCDs
3. Understanding the benefits of three types of exercise (i.e., aerobic, strength building and stretching)
4. Raising curiosity on how much and what type of physical activity should an adolescent do to prevent NCDs

Issue 3: Obesity

1. Demonstrating the concept of normal weight and obesity
2. Enabling to calculate one’s Body Mass Index (BMI) status
3. Understanding and identifying the potential causes of obesity
4. Understanding the health consequence of childhood obesity
5. Raising curiosity on what should be the ideal weight to stay fit and how to achieve it

Issue 4: Smoking

1. Understanding the concept and the effect of active and passive smoking
2. Understanding the relation of smoking with NCDs
3. Identifying the common reasons to start smoking at adolescent age
4. Raising curiosity on how to avoid starting smoking or how to quit smoking or how to reduce passive smoking

Issue 5: NCDs

1. Understanding the basic pathology and aetiology of NCDs with special emphasis on atherosclerosis, stroke, heart disease, diabetes and hypertension
2. Understanding and identifying the effect of lifestyle behavior on NCDs
3. Recognizing the preventive ways of NCDs
4. Raising curiosity on how can and to what extent should one apply the preventive ways to avert NCDs

Issue 6: Preventive ways

1. Understanding the daily requirements of different foods to prevent NCDs
2. Enabling to make one’s own platter
3. Understanding the recommended physical activity requirements at adolescent age
4. Enabling to perform aerobic and stretching exercise
5. Understanding the ways to maintain normal body weight
6. Making healthy choices for lifestyle behavior and setting goals

The intervention will be delivered through a health promotion session using multi-media projector, flip paper and pen and leaflets. Each session will be of 2 h duration. A sequential framework of the intervention conceptualizing from a standard MI training manual [29] and SCT theory [18] will be used to train the facilitators.

Here is the sequential framework of the intervention workout:

**Table:** 

Concept	Objectives	Illustration with example
Creation of motivational environment	✓ Creating a collaborative environment that evoke motivation. ✓ Giving honour to students’ autonomy and self-direction.	• Facilitators will be trained to be supportive, suspend an authoritarian role, explore capacity of the students rather than incapacity. e.g. facilitators will invite the participants to correlate different types of foods with different health status in pictorial view. Here facilitators will show a sincere interest in the students’ experiences and perspectives.
Establish rapport and express empathy	Explaining the aim of the session. Showing concern about their health. Making positive environment where the students feel free to talk about themselves.	Facilitators will ask the students to talk about their general perception regarding some common NCDs and their risk factors. Facilitators will also invite them to share their experience with NCD patients in their family. These conversations will be non-judgmental and with proper empathy.
Self-exploration of behavior	Letting students explore their experience, knowledge and ambivalence in a supportive and facilitative atmosphere.	• Facilitators will practice making open ended relevant questions, avoiding uncomfortable ones, e.g. facilitators will ask students about eating vegetables regularly. Facilitators may ask them why they eat vegetables every day or why not, and what will be the perceived health consequences in both cases without making any judgement. The co-facilitator will jot the answers briefly down a flip paper. Then, facilitators will summarize responses and give affirmation.
Practical knowledge sharing	Learning by observation.	Facilitators will show the link between sugar sweetened beverages and obesity, using pictures and short videos. Facilitators will show the consequences of different NCDs such as diabetes through videos.
Developing discrepancy	Understanding the difference between current behavior and its consequences with the desired health status	Facilitators will recapitulate their current salty food consumption and help them recognizing the discrepancy between their current behavior and their desire to stay free from cardiovascular diseases.
Recognizing change talk and sustain talk	✓ Identifying change talk and commitment language that signals movement in the direction of behaviour change, as well as sustain talk.	• Facilitators will try to recognize this kind of talks “maybe my soft drink consumption will put me in trouble” or “now I am worried about my weight” and will give affirmation to these change talk.
Roll with resistance	Rolling with the conflict of students rather than opposing	Some students may oppose or show disagreement about changing behavior. Facilitators will not compete with them, rather take another approach to make the issue clearer to the students. Facilitators will respond to Students’ sustain talk and resistance in a manner that reflects respect without reinforcing the desired behavior.
Skill development	Learning to perform new behaviors to overcome barriers towards healthy lifestyle through observation and peer modeling	Facilitators will recruit some volunteer role models from the students and teach them how to perform some effective free hand stretching exercises in a resource poor setting. Students will observe the models and adopt the best suit for themselves.
Developing Change Plan	✓ Making the transition into practice.	Facilitators will encourage the students to explore their barriers to be physically active and try to discover their own potential solutions giving emphasis on timing and negotiation skill.
Improve self-evaluated outcome expectancy	Improving hopes and beliefs about the consequences of desired behavior.	Facilitators will help students evaluate and improve expectations about smoke free life or physically active life.
Consolidatingcommitments	✓ Eliciting strength of changing commitment	• Facilitators will highlight students’ changing commitment to reinforce them. Facilitators will give emphasis on specific implementation intentions.
Support self-efficacy	Supporting the belief in personal ability to adopt healthy behavior	Students will be asked to express their self-efficacy about doing physical activity in an efficacy measuring scale and write down individual plan to acquire adequate daily physical activity.
Verbal persuasion	Strengthening self-efficacy	Facilitators will encourage students with culturally accepted and stimulating words e.g. facilitators may use trending words of this age.
Setting up self-regulatory ways	Constructing own goal and self-monitoring ways to achieve the goal	Facilitators will encourage and support students to build personal goal to consume at least five servings of fruits per day and to set self-monitoring ways to achieve the goal.

## Discussion

This intervention aims to motivate adolescents towards positive health behaviors. Motivation can be postulated as the reason to action. Motivation can change individual’s perception and thus prepare oneself to accept the changing process. MI is one of the most thoroughly used and successful method for counseling aimed at promoting health behavior. In this study, students will be motivated using a few MI principles [30, 31] to trigger their concern about their own health and then help them going through the changing process. Although MI was designed and used in clinical settings extensively, it can be successfully applied in school settings too [32].

MI is mostly used in alcohol and drug abuse cases [33], and in case of smoking [34]. However, MI is also used in other health behavior changes as well [35]. Originally, MI was designed to be delivered in person but, MI delivered in a group also showed significant success in making desired change [36]. In this study, the principles of MI will be applied to counsel a group of students in school setting, who will be homogenous in terms of age, sex, socio-economic status and risk behavior status. Thus, the true essence of MI principles is expected to be preserved. The facilitators will be selected and trained meticulously on MI, according to the guidelines developed by Miller (1995), to maintain the standard of MI portion of the intervention.

Once the students are motivated enough to show their interest in healthy behavior, the process of negotiating change and strengthening commitment will be started by providing information. The information will be delivered in a structured way based on the principles of Bandura’s Social Cognitive Theory (SCT) [12]. The objective is to improve psychological determinants of behavior through observational learning. This intervention will emphasize supporting self-efficacy that is the belief in performance which brings the desire change [13]. As for example, if self-efficacy of a student about healthy diet is high, it means he believes that he can manage healthy eating at any situation. In this intervention, focus will be given on improving this self-efficacy through sharing experience, modeling and through verbal persuasion using some trend words, like “you are Bangladesh, you can do it”. In addition, education about NCDs and its risk factors will be given in such a way that allows them to reflect, letting them develop, and evaluate their own outcome expectancy which is, another behavior changing component of Bandura’s theory [18].

This intervention is not aimed to bring any environmental change. Yet, this intervention will demonstrate choices and make them skilled, thus empower them to pick the most suitable choice for themselves. As for example, they will be taught with a range of free hand exercises by role modeling and by making some role models among themselves. Now as they are mastered on several exercises, they can do any of them at their convenience, to acquire the daily physical activity recommendation. Leaflets on healthy diet, physical activity and anti-tobacco message will be provided to take home, which is expected to improve the social support to some extent. These will eventually improve their self-efficacy and self-regulatory capacity and thus influence health behavior.

An intervention is judged by its cost-effectiveness when referring for mass population. Meta-analysis on this issue found that group intervention is the least expensive yet most cost-effective interventional way among the adolescents [37]. This proposed intervention is designed for group and therefore, is more realistic in the low socio-economic context like Bangladesh to deliver the intervention to utilize the limited resources best. Thus, upon its effectiveness being proven, this intervention can be advocated to policies.

Any behavior to persist long-term requires social support, supportive environments, and periodic reinforcement [18]. Social engagement or environmental reformation is beyond the scope of this study and the long-term reinforcement of the student. With limited time and financial support, present study is expected to gather valuable knowledge about development of effective NCDs prevention intervention among adolescents of Bangladesh.

## Additional files

Additional file 1: Study design (Stages of the study) (DOC 33 kb)

Additional file 2: Questionnaire (in English) (DOC 253 kb)

Additional file 3: SPIRIT Checklist (DOC 131 kb)

## Data Availability

Data sharing is not applicable to this article as this is a protocol paper.

## Abbreviations

BMI: Body mass index
BSMMU: Bangabandhu Sheikh Mujib Medical University
MI: Motivational interview
NCD: Non-communicable disease
SCT: Social cognitive theory
SSB: Sugar sweetened beverage
STEPs: Stepwise Surveillance for NCD risk factors
TREND: Transparent reporting of evaluations with non-randomized designs
