# Supplementary material for: Towards reducing behavioral risk factors of non-communicable diseases among adolescents: protocol for a school-based health education program in Bangladesh
Source: BMC Public Health. 2019 Jul 25;19:1002. doi: 10.1186/s12889-019-7229-8 (PMC6659286; doi:10.1186/s12889-019-7229-8)
Supplement: Supplementary file 1 — Study design (Stages of the study) (DOC 33 kb) [file 12889_2019_7229_MOESM1_ESM.doc]

Additional file 1

Figure
