# Supplementary material for: Towards reducing behavioral risk factors of non-communicable diseases among adolescents: protocol for a school-based health education program in Bangladesh
Source: BMC Public Health. 2019 Jul 25;19:1002. doi: 10.1186/s12889-019-7229-8 (PMC6659286; doi:10.1186/s12889-019-7229-8)
Supplement: Supplementary file 2 — Questionnaire (in English) (DOC 253 kb) [file 12889_2019_7229_MOESM2_ESM.doc]

**Study title: An intervention program to reduce non-communicable diseases related behavioral risk factors among adolescents in institutional settings of Bangladesh**

**Principal investigator: Dr. Md. Khalequzzaman**

**Associate Professor, Department of Public Health and Informatics,**

**Bangabandhu Sheikh Mujib Medical University, Shahbag, Dhaka.**

**Participant’s-**

**Class .............**

**Section……...**

**Roll no............**

**(Tick the answers you think most appropriate)**

**Conducted by:**

**Chi Research and Infotec Ltd.**

1. **General Knowledge and Attitudes related to Non- Communicable Diseases**

|  | **Question** | **Response** | **Ans. code** | **Ques.**  **Code** |
| --- | --- | --- | --- | --- |
| 1 | Can Non-communicable diseases spread between people? | Cannot spread  Can spread  I don’t know | 1  2  3 | K1 |
| 2 | How much dangerous are non-communicable diseases? | Ver much dangerous  Quite dangerous  Not at all | 3  2  1 | A1 |
| 3 | What do you know about the prevalence of Non-communicable diseases among Bangladeshis? | These are common  These are not common  I don’t know | 1  2  3 | K2 |
| *Non-communicable diseases are a group of diseases that include heart disease, diabetes, hypertension, chronic respiratory diseases, cancers etc.* | | | | |

**2. Knowledge, Attitudes and Practices on NCD Behavioural Risk Factors (RF)**

| **Dietary habit** | | |  |  |
| --- | --- | --- | --- | --- |
| 4 | How many days do you eat fruits in last 7 days? (e.g. banana, tormuj, bangi, mango, apple, orange, pineapple, boroi, amra etc.) | days |  | P1 |
| 5 | How many servings of fruit do you eat on one of those days? | Servings |  | P2 |
| 6 | Do you think you eat adequate amount of fruits every day? | Yes  No  I don’t know | 1  2  3 | A2 |
| 7 | How much important it is to you to eat fruits every day? | Very much important  Quite important  Not at all | 3  2  1 | A3 |
| 8 | What are the benefits of eating fruits every day?  (Multiple answers acceptable) | It keeps good health  It prevents NCD  It prevents constipation  It fulfils nutritional need  Others (Please mention) …………. | 1  2  3  4  77 | K3 |
| 9 | What are the reasons of not eating fruits every day?  (Multiple answers acceptable) | High price  Not so available  Fruits are not accessible at home everyday  No one eats fruit everyday around me  Others (Please mention) …………. | 1  2  3  4  77 | B1 |
| 10 | Will you eat fruits every day from now? | Yes  No  Not sure | 1  2  3 | B2 |
| 11 | How can you increase your fruits consumption?  (Multiple answers acceptable) | Ask parents to buy fruits everyday  Eat fruits instead of unhealthy snacks  Eat fruits in every meal  Tell others to eat fruits  Others (Please mention) …………. | 1  2  3  4  77 | B3 |
| 12 | Did anyone tell you or encourage you to eat fruits every day? Who? | Parents  Teachers  Friends  Health workers  Others (Please mention) ………….    No one told me | 1  2  3  4  77  5 | B4 |
| 13 | How many days do you eat vegetables in last 7 days? e.g. Green and coloured leafy vegetables, bottle Guard, Papaya, tomato, cauliflower, cabbage, beans, brinjal, ladies finger, cucumber or chichinga etc.  (Not potato, rice or other cereals) | days |  | P3 |
| 14 | How many servings of vegetables do you eat on one of those days? | Servings |  | P4 |
| 15 | Do you think you eat adequate amount of vegetables every day? | Yes  No  I don’t know | 1  2  3 | A4 |
| 16 | How much important it is to you to eat vegetables every day? | Very much important  Quite important  Not at all | 3  2  1 | A5 |
| 17 | What are the benefits of eating vegetables every day?  (Multiple answers acceptable) | It keeps good health  It prevents non-communicable disease  It prevents constipation  It fulfils nutritional need  Others (Please mention) …………. | 1  2  3  4  77 | K4 |
| 18 | What are the reasons of not eating vegetables every day?  (Multiple answers acceptable) | High price  Not so available  Fruits are not accessible at home everyday  No one eats fruit everyday around me  Others (Please mention) …………. | 1  2  3  4  77 | B5 |
| 19 | Will you eat vegetables every day from now? | Yes  No  Not sure | 1  2  3 | B6 |
| 20 | How can you increase your vegetables consumption?  (Multiple answers acceptable) | Ask parents to buy fruits everyday  Eat fruits instead of unhealthy snacks  Eat fruits in every meal  Tell others to eat fruits  Others (Please mention) …………. | 1  2  3  4  77 | B7 |
| 21 | Did anyone tell you or encourage you to eat vegetables every day? Who? | Parents  Teachers  Friends  Health workers  Others (Please mention) ………….    No one told me | 1  2  3  4  77  5 | B8 |
| 22 | How many days do you consume soft drinks in a typical week? (e.g. Pepsi, coca cola, sprite, 7-up, fanta, mirinda, RC cola, mojo etc.) | days |  | P5 |
| 23 | How much harmful it is to you to drink sugar sweetened beverages every day? | Very much harmful  Quite harmful  Not at all harmful | 3  2  1 | A6 |
| 24 | How many days do you consume energy drinks in a typical week? (e.g.tiger, speed, royal etc.) | days |  | P6 |
| 25 | Do you ever think about how much salt you should have in your diet every day? | Yes, I thought  No, I never thought | 1  2 | B9 |
| 26 | Do you take extra salt on your plate? | Always  Sometimes  Never | 1  2  3 | P7 |
| 27 | What is your opinion about taking extra salt during meal? | Harmful for health  Good for health  Not related to health | 1  2  3 | A7 |
| **Physical activity** | | | |  |
| 28 | During the past 7 days, on how many days were you physically active for a total of at least 60 minutes or more per day? ADD UP ALL THE TIME YOU SPENT IN ANY KIND OF PHYSICAL ACTIVITY EACH DAY.  (Physical activity means exercise, walking, running, playing in field, swimming, cycling, working in the field etc) | 0 days  1 day  2 days  3 days  4 days  5 days  6 days  7 days | 1  2  3  4  5  6  7  8 | P8 |
| 29 | During the past 7 days, on how many days did you walk or ride a bicycle to or from school/ coaching/ market/ any other place? | 0 days  1 day  2 days  3 days  4 days  5 days  6 days  7 days | 1  2  3  4  5  6  7  8 | P9 |
| 30 | During this school year, on how many days did you go to physical education (PE) class each week? | 0 days  1 day  2 days  3 days  4 days  5 or more days | 1  2  3  4  5  6 | P10 |
| 31 | During the past 7 days, on how many days did you do exercises to strengthen or tone your muscles, such as push-ups, sit-ups, or weight lifting?  (See the pictures if needed) | 0 days  1 day  2 days  3 days  4 days  5 days  6 days  7 days | 1  2  3  4  5  6  7  8 | P11 |
| 32 | During the past 7 days, on how many days did you do muscle stretching exercises such as touching toe while bending your waist or stretching your legs?  (See the pictures if needed) | 0 days  1 day  2 days  3 days  4 days  5 days  6 days  7 days | 1  2  3  4  5  6  7  8 | P12 |
| 33 | Do you think you do adequate physical activity every day? | Yes  No  Don’t know | 1  2  3 | A8 |
| 34 | How much important it is to you to do physical exercise every day? | Very much important  Quite important  Not at all | 3  2  1 | A9 |
| 35 | What are the health benefits of doing physical activity every day?  (Multiple answer accepted) | It keeps body healthy  Prevents non-communicable disease  Increases brain efficacy  Reduces chance of heart disease  Reduces chance of diabetes  Increases working capacity  Others (Please specify) ...... | 1  2  3  4  5  6  77 | K5 |
| 36 | In your opinion how often should a person at your age do exercise to stay healthy? | Monthly  Twice a month  Once a week  2-4 times per week  5-6 times per week  Every day  Others (Please specify) ...... | 1  2  3  4  5  6  77 | K6 |
| 37 | What is the main reasons of not doing physical activity every day?  (From the following options, choose one.) | Time constraint  Too expensive  They don’t know how  They don’t want to  They think there is no need  Scarcity of place  Other (specify)__________ | **1**  **2**  **3**  **4**  **5**  **6**  **77** | B10 |
| 38 | From now will you do physical activity every day? | Yes  No  Not sure | **1**  **2**  **3** | B11 |
| 39 | How can you increase your physical activity every day?  (You can choose multiple answers) | Play outdoor game every day  Attend physical exercise class  Go school by cycling or walking  Tell others to do physical activity everyday  Others (specify) ......... | **1**  **2**  **3**  **4**  **77** | B12 |
| 40 | Did anyone tell you or encourage you to do physical activity every day? Who? | Parents  Teachers  Friends  Health workers  Others (Please mention) ………….    No one told me | **1**  **2**  **3**  **4**  **77**  **5** | B13 |
| 41 | How much time do you usually spend sitting or reclining on a typical day?  (e.g. watching television, doing computer work, playing video game, chatting with friends, sewing etc)  (Time spending at classroom or at home doing homework is not included) | Less than 1 hour a day  1-2 hours a day  3-4 hours a day  5-6 hours a day  7-8 hours a day  More than 8 hours a day | **1**  **2**  **3**  **4**  **5**  **6** | P13 |
| 42 | How much time do you spend sleeping in a typical day?  (Add night sleep and day nap time) | 4 hours or less  5 hours  6 hours  7 hours  8 hours  9 hours  10 hours or more | **1**  **2**  **3**  **4**  **5**  **6**  **7** | P14 |
| **Tobacco use** | | | |  |
| 43 | How old were you when you first tried a cigarette? | I have never smoked cigarettes  7 years old or younger  8 or 9 years old  10 or 11 years old  12 or 13 years old  14 or 15 years old  16 or 17 years old  18 years old or older | 1  2  3  4  5  6  7  8 | P15 |
| 44 | During the past 30 days, on how many days did you smoke cigarettes? | 0 days  1 or 2 days  3 to 5 days  6 to 9 days  10 to 19 days  20 to 29 days  All 30 days | 1  2  3  4  5  6  7 | P16 |
| 45 | During the past 30 days, on how many days did you use any tobacco products other than cigarettes, such as biri, jarda, tobacco leaf, noshi, gul, khoni, khoyer or shisha? | 0 days  1 or 2 days  3 to 5 days  6 to 9 days  10 to 19 days  20 to 29 days  All 30 days | 1  2  3  4  5  6  7 | P17 |
| 46 | During the past 12 months, have you ever tried to stop smoking cigarettes? | Yes  No  I have never smoked cigarettes  I did not smoke cigarettes during the past 12 months | 1  2  3  4 | P18 |
| 47 | During the last 30 days, how many times you tried to stop smoking? | ................... times |  | P19 |
| 48 | During the past 7 days, on how many days have people smoked in your presence? | 0 days  1 or 2 days  3 or 4 days  5 or 6 days  All 7 days | 1  2  3  4  5 | P20 |
| 49 | Which of your parents or guardians use any form of tobacco e.g. cigarettes, biri, jarda, tobacco leaf, noshi, gul, khoni, khoyer or shisha?? | My father or male guardian  My mother or female guardian  Both  Neither | 1  2  3  4 | P21 |
| 50 | Do you think smoking is harmful for health? | Yes  No  I don’t know | 1  2  3 | K7 |
| 51 | How much do you have to smoke for it to harm your health? | Any smoke harms health  Smoking at least once a week  Only daily smoking is harmful  Only a packet of cigarettes or more per day is harmful | 1  2  3  4 | K8 |
| 52 | How much harmful smoking is for lungs? | Very much harmful  Quite harmful  Not at all | 3  2  1 | A10 |
| 53 | How much harmful smoking is for heart? | Very much harmful  Quite harmful  Not at all | 3  2  1 | A11 |
| 54 | How much harmful smoking is for brain? | Very much harmful  Quite harmful  Not at all | 3  2  1 | A12 |
| 55 | Do you think smoking around others could affect their health? | Yes  No  I don’t know | 1  2 | K9 |
| 56 | Do you mind if people smoke in your home? | Don’t mind  I do mind, but I allow it  I don’t allow it | 1  2  3 | A13 |
| 57 | What will be the benefit of not smoking?  (Multiple answer acceptable) | Money and time will be saved  Keeps good health  Prevents NCD  Increase brain efficacy  Reduces chance of heart disease  Reduces chance of lung cancer  Increase working capacity  Others (Specify) ………... | 1  2  3  4  5  6  7  77 | K10 |
| 58 | In your opinion why does one at your age smoke?  (Choose any one) | With friends/ Peer pressure  Watching elder of the family  To prove oneself adult  To forget grief  It feels good to smoke  From curiosity  Others (Specify) ………….. | 1  2  3  4  5  6  77 | B14 |
| 59 | Did anyone tell you or encourage you not to do smoking? Who?  (Multiple answer accepted) | Parents  Teachers  Friends  Health workers  Others (Please mention) ………….    No one told me | **1**  **2**  **3**  **4**  **77**  **5** | B15 |
| **Alcohol and Drugs** | | | |  |
| 60 | During the past 30 days, on how many days did you have at least one drink containing alcohol? (Bear, vodka, waine, bangla mod, cholai mod etc) | 0 days  1 or 2 days  3 to 5 days  6 to 9 days  10 to 19 days  20 to 29 days  All 30 days | 1  2  3  4  5  6  7 | P22 |
| **61** | Have you ever taken adiictive drugs? (Tick what you took)  (Multiple answer accepted) | Ganja  Yaba  Heroin  Fensidil  Others (specify)………  No, never take any | 1  2  3  4  77  5 | P23 |
| Weight | | | |  |
| **62** | Have you weighed yourself in the last 6 months? | Yes  No | 1  2 | P24 |
| 63 | Regarding your body weight, what do you feel you are? | Underweight  Normal weight  Overweight  Very overweight | *1*  *2*  *3*  *4* | A14 |
| **64** | How much important it is to you to maintain normal weight to stay healthy? | Very much important  Quite mportant  Not at all important | 3  2  1 | A15 |
| High Blood pressure | | | | |
| 65 | How much do you know about “blood pressure”? | Nothing at all  I have only heard the term before  I know about it | 1  2  3 | K11 |
| 66 | Do you think it is important to check blood pressure regularly? | Yes  No  Don’t know | 1  2  3 | A16 |
| 67 | How would eating food with a lot of salt affect your blood pressure? | Raise blood pressure  Lower blood pressure  Don’t know | 1  2  3 | K12 |
| 68 | Does high blood pressure can cause health problems? | Yes  No  Don’t know | 1  2  3 | K13 |
| **69** | Which one is effective to prevent high blood pressure?  (Multiple answer accepted) | Medication  Losing weight  Changing diet pattern  Physical exercise  Others (specify)………  I don’t know | 1  2  3  4  77  6 | K14 |

**3. KAP about NCD**

| Questions | | Answers | | | | Code |
| --- | --- | --- | --- | --- | --- | --- |
| **Cardiovascular diseases** | | | | | | |
| 70 | How much do you know about “heart disease”? | Nothing at all  I have only heard the term before  I know about it | 1  2  3 | | K15 | |
| 71 | Which of the following things you think would increase someone’s chances of getting cardiovascular diseases?  (Multiple answer accepted) | Smoking  High blood pressure  Excess weight  Older age  Fatty food  Salty food | 1  2  3  4  5  6 | K16 | | |
| 72 | How much do you know about “Stroke”? | Nothing at all  I have only heard the term before  I know about it | 1  2  3 | K17 | | |
| 73 | Which organ is affected by stroke? | Heart  Brain  I don’t know | 1  2  3 | K18 | | |
| 74 | Which of the following things you think would increase someone’s chances of getting stroke? (Multiple answer accepted) | Smoking  High blood pressure  Excess weight  Older age  Fatty food  Salty food  Others (Specify) …….  I don’t know | 1  2  3  4  5  6  77  7 | K19 | | |
| 75 | Do you think we can prevent cardiovascular disease and stroke? | Yes  No  Don’t know | 1  2  3 | K20 | | |
| 76 | How much do you know about “Cervical cancer”? | Nothing at all  I have only heard the term before  I know about it | 1  2  3 | K21 | | |
| 77 | Do you know if cervical cancer can be prevented by vaccine? | Yes  No  Don’t know | 1  2  3 | K22 | | |
| **Diabetes** | | | | | | |
| 78 | How much do you know about “Diabetes”? | Nothing at all  I have only heard the term before  I know about it | 1  2  3 | K23 | | |
| 79 | Do you think diabetes is preventable? | Yes  No  Don’t know | 1  2  3 | K24 | | |
| 80 | Can you think of things a person can do to reduce their chances of getting diabetes?  (Multiple answer accepted) | Changing dietary habit  Medication  Physical activity  Losing weight  Quitting smoking  I don’t know  Diabetes can’t be prevented by any means  Others (Specify) ……. | 1  2  3  4  5  6  7  77 | K25 | | |
| 81 | Has a health worker ever spoken to you about how you can prevent diabetes? | Yes  No  I don’t know/ I can’t remember | 1  2  3 | P25 | | |

**4. Socio-demographic Information**

| SN | Questions | | Answers | Ans.Code | Ques. Code | |
| --- | --- | --- | --- | --- | --- | --- |
| 82 | | What is your gender? | Male  Female | 1  2 | | SD1 |
| 83 | | How old are you? | └─┴─┘ Years |  | | SD2 |
| 84 | | Which of the following best describes your father’s main occupation in last twelve months?  (Tick any one) | Government employee (Officer)  Government employee (Others)  Non-government employee (Officer)  Non-government employee (Others)  Business (Large)  Business (Small)  Housewife/ Homemaker  Household worker  Farming and Land owner  Agricultural worker  Factory worker  Building worker  Garments worker  Garage worker  Home commerce worker Rickshaw/van/push cart driver Poultry  Student  Unemployed, able to work  Unemployed, unable to work  Refused  Other (specify)…… | 1  2  3  4  5  6  7  8  9  10  11  12  13  14  15  16  17  18  19  20  99  77 | | SD3 |
| 85 | | Which of the following best describes your mother’s main occupation in last twelve months?    (Tick any one) | Government employee (Officer)  Government employee (Others)  Non-government employee (Officer)  Non-government employee (Others)  Business (Large)  Business (Small)  Housewife/ Homemaker  Household worker  Farming and Land owner  Agricultural worker  Factory worker  Building worker  Garments worker  Garage worker  Home commerce worker Rickshaw/van/push cart driver Poultry  Student  Unemployed, able to work  Unemployed, unable to work Refused  Other (specify)…… | 1  2  3  4  5  6  7  8  9  10  11  12  13  14  15  16  17  18  19  20  99  77 | | SD4 |
| 86 | | What is the **highest level of formal education** your father has completed?  (Tick any one) | No formal schooling  Less than primary school  Primary school completed  Secondary school completed  Higher Secondary school completed  College/University completed  Post graduate degree completed  Refused | 1  2  3  4  5  6  7  99 | | SD5 |
| 87 | | What is the **highest level of formal education** your mother has completed?  (Tick any one) | No formal schooling  Less than primary school  Primary school completed  Secondary school completed  Higher Secondary school completed  College/University completed  Post graduate degree completed  Refused | 1  2  3  4  5  6  7  99 | | SD6 |
| 88 | | Does your father or mother or any member of your family has any of the following diseases? Please specify.  (Multiple answer accepted) | Cardiovascular disease  Diabetes  High pressure  Stroke  Cancer    No one | 1  2  3  4  5  6 | | SD7 |
| 89 | | Which of the following items are present at your household? (Multiple answer accepted) | Electricity  Flash toilet  TNT phone (Land phone)  Mobile phone  Television  Radio  Refrigarator  Air conditioner  IPS/ Generator  Rickshaw/van  Private car  Scooter/ motor cycle/ Autobike/ Tempo/ CNG  Bicycle  Washing Machine  Almira  Table  Chair/bench  Clock/watch  Bed/cot  Sewing machine  Computer/laptop | 1  2  3  4  5  6  7  8  9  10  11  12  13  14  15  16  17  18  19  20  21 | | SD8 |
| 90 | | Does your educational institute have a playground? | Yes  No | 1  2 | | SD9 |

**Thank you**

***(Data collectors will complete this section)***

**5. Physical measurement**

| **SN** | **Variables** | **Value** | | | **Code** |
| --- | --- | --- | --- | --- | --- |
| **91** | Height (cm) |  | | | PM1 |
| **92** | Weight (kg) |  | | | PM2 |
| **93** | Waist circumference (cm) |  | | | PM3 |
| **94** | Hip circumference (cm) |  | | | PM4 |
| **95** | Pulse (per minute) |  | | | PM5 |
| **96** | Blood pressure (systolic) | 1st | 2nd | 3rd | PM6 |
| 97 | Blood pressure (diastolic) | 1st | 2nd | 3rd | PM7 |

**Sign of data collector:**

**Date:**
