# Supplementary material for: Towards reducing behavioral risk factors of non-communicable diseases among adolescents: protocol for a school-based health education program in Bangladesh
Source: BMC Public Health. 2019 Jul 25;19:1002. doi: 10.1186/s12889-019-7229-8 (PMC6659286; doi:10.1186/s12889-019-7229-8)
Supplement: Supplementary file 3 — SPIRIT Checklist (DOC 131 kb) [file 12889_2019_7229_MOESM3_ESM.doc]

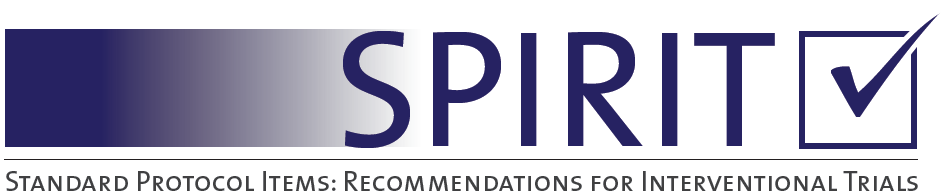


SPIRIT 2013 Checklist: Recommended items to address in a clinical trial protocol and related documents*

| Section/item | Item No | Description | Addressed on page number |
| --- | --- | --- | --- |
| **Administrative information** | | |  |
| Title | 1 | Descriptive title identifying the study design, population, interventions, and, if applicable, trial acronym | Page 1 |
| Trial registration | 2a | Trial identifier and registry name. If not yet registered, name of intended registry | ClinicaTrials.gov (registration is in process) |
|  | 2b | All items from the World Health Organization Trial Registration Data Set | Primary registry and trial identifying number: Registration is in process with ClinicaTrials.gov  Date of registration in primary registry: Registration is in process  Secondary identifying numbers: N/A  Source(s) of monetary or material support: Directorate General of Health Services, Ministry of Health & Family Welfare, Mohakhali, Dhaka-1212.  Primary sponsor: Chi Research and Infotec Ltd.  Secondary sponsor(s): N/A  Contact for public queries: Marium Salwa (email: mariumsalwa@gmail.com)  Contact for scientific queries: Marium Salwa (email: [mariumsalwa@gmail.com](mailto:mariumsalwa@gmail.com))  Public title: An intervention program to reduce NCD related behavioral risk factor among adolescents in institutional settings of Bangladesh  Scientific title: Towards reducing behavioral risk factors of non-communicable diseases among adolescents: Protocol for a school-based health education program in Bangladesh  Countries of recruitment: Bangladesh  Health condition(s) or problem(s) studied: Risk behaviors- unhealthy diet, physical inactivity, smoking and alcohol use, obesity  Intervention(s): Health education (lectures and classroom activities) (Page 11)  Key inclusion and exclusion criteria: Participant recruitment and data collection (page 5)  Study type: Interventional (Before-after design) (page 5)  Date of first enrolment: April 2018  Target sample size: 548  Recruitment status: Recruited  Primary outcome(s): Knowledge, attitude and practice on NCD behavioral risk factors (page 7)  Key secondary outcomes: Body Mass Index, Blood Pressure (page 7) |
| Protocol version | 3 | Date and version identifier | N/A |
| Funding | 4 | Sources and types of financial, material, and other support | Page 21 (Funding) |
| Roles and responsibilities | 5a | Names, affiliations, and roles of protocol contributors | Page1 and Page 20 (Authors’ contributions) |
|  | 5b | Name and contact information for the trial sponsor | Chi Research & Infotec Ltd.  Khairunnesa Mansion (3^rd^ Floor), 234-D, New Elephant Road, Dhaka-1212  Email: crilbd@gmail.com |
|  | 5c | Role of study sponsor and funders, if any, in study design; collection, management, analysis, and interpretation of data; writing of the report; and the decision to submit the report for publication, including whether they will have ultimate authority over any of these activities | Page 21 (Funding) |
|  | 5d | Composition, roles, and responsibilities of the coordinating centre, steering committee, endpoint adjudication committee, data management team, and other individuals or groups overseeing the trial, if applicable (see Item 21a for data monitoring committee) | MS: Coordination of the study  MK: Principal Investigator  MAH, AAM, MRB, SRC: Steering committee members |
| Introduction |  |  |  |
| Background and rationale | 6a | Description of research question and justification for undertaking the trial, including summary of relevant studies (published and unpublished) examining benefits and harms for each intervention | Page 4 and 5 (Background) |
|  | 6b | Explanation for choice of comparators | Page 4 and 5 (Background) |
| Objectives | 7 | Specific objectives or hypotheses | Page 5 and 6 (Objectives) |
| Trial design | 8 | Description of trial design including type of trial (eg, parallel group, crossover, factorial, single group), allocation ratio, and framework (eg, superiority, equivalence, noninferiority, exploratory) | Page 6 (Study design and setting) |
| Methods: Participants, interventions, and outcomes | | |  |
| Study setting | 9 | Description of study settings (eg, community clinic, academic hospital) and list of countries where data will be collected. Reference to where list of study sites can be obtained | Page 6 (Study design and setting) |
| Eligibility criteria | 10 | Inclusion and exclusion criteria for participants. If applicable, eligibility criteria for study centres and individuals who will perform the interventions (eg, surgeons, psychotherapists) | Page 6 (Participant recruitment and data collection) |
| Interventions | 11a | Interventions for each group with sufficient detail to allow replication, including how and when they will be administered | Page 6 (Participant recruitment and data collection) |
|  | 11b | Criteria for discontinuing or modifying allocated interventions for a given trial participant (eg, drug dose change in response to harms, participant request, or improving/worsening disease) | N/A |
|  | 11c | Strategies to improve adherence to intervention protocols, and any procedures for monitoring adherence (eg, drug tablet return, laboratory tests) | Page 10 (Quality control measurement) |
|  | 11d | Relevant concomitant care and interventions that are permitted or prohibited during the trial | N/A |
| Outcomes | 12 | Primary, secondary, and other outcomes, including the specific measurement variable (eg, systolic blood pressure), analysis metric (eg, change from baseline, final value, time to event), method of aggregation (eg, median, proportion), and time point for each outcome. Explanation of the clinical relevance of chosen efficacy and harm outcomes is strongly recommended | Page 8 and 9 (Outcome variables) |
| Participant timeline | 13 | Time schedule of enrolment, interventions (including any run-ins and washouts), assessments, and visits for participants. A schematic diagram is highly recommended (see Figure) | Figure 1 (Additional file 1) |
| Sample size | 14 | Estimated number of participants needed to achieve study objectives and how it was determined, including clinical and statistical assumptions supporting any sample size calculations | Page 6 (Participant recruitment and data collection) |
| Recruitment | 15 | Strategies for achieving adequate participant enrolment to reach target sample size | Page 6 (Participant recruitment and data collection) |
| **Methods: Assignment of interventions (for controlled trials)** | | |  |
| Allocation: |  |  |  |
| Sequence generation | 16a | Method of generating the allocation sequence (eg, computer-generated random numbers), and list of any factors for stratification. To reduce predictability of a random sequence, details of any planned restriction (eg, blocking) should be provided in a separate document that is unavailable to those who enrol participants or assign interventions | Page 6 (Study design and setting, Participant recruitment and data collection) |
| Allocation concealment mechanism | 16b | Mechanism of implementing the allocation sequence (e.g. central telephone; sequentially numbered, opaque, sealed envelopes), describing any steps to conceal the sequence until interventions are assigned | Page 6 (Study design and setting, Participant recruitment and data collection) |
| Implementation | 16c | Who will generate the allocation sequence, who will enrol participants, and who will assign participants to interventions | Page 10 (Quality control measurement) |
| Blinding (masking) | 17a | Who will be blinded after assignment to interventions (eg, trial participants, care providers, outcome assessors, data analysts), and how | Page 6 (Participant recruitment and data collection) |
|  | 17b | If blinded, circumstances under which unblinding is permissible, and procedure for revealing a participant’s allocated intervention during the trial | N/A |
| **Methods: Data collection, management, and analysis** | | |  |
| Data collection methods | 18a | Plans for assessment and collection of outcome, baseline, and other trial data, including any related processes to promote data quality (eg, duplicate measurements, training of assessors) and a description of study instruments (eg, questionnaires, laboratory tests) along with their reliability and validity, if known. Reference to where data collection forms can be found, if not in the protocol | Page10 (Quality control measurement) |
|  | 18b | Plans to promote participant retention and complete follow-up, including list of any outcome data to be collected for participants who discontinue or deviate from intervention protocols | Page 6 (Participant recruitment and data collection) |
| Data management | 19 | Plans for data entry, coding, security, and storage, including any related processes to promote data quality (eg, double data entry; range checks for data values). Reference to where details of data management procedures can be found, if not in the protocol | Page 10 (Data management plan) |
| Statistical methods | 20a | Statistical methods for analysing primary and secondary outcomes. Reference to where other details of the statistical analysis plan can be found, if not in the protocol | Page 10 (Statistical analysis plan) |
|  | 20b | Methods for any additional analyses (eg, subgroup and adjusted analyses) | Page 10 (Statistical analysis plan) |
|  | 20c | Definition of analysis population relating to protocol non-adherence (eg, as randomised analysis), and any statistical methods to handle missing data (eg, multiple imputation) | N/A |
| **Methods: Monitoring** | | |  |
| Data monitoring | 21a | Composition of data monitoring committee (DMC); summary of its role and reporting structure; statement of whether it is independent from the sponsor and competing interests; and reference to where further details about its charter can be found, if not in the protocol. Alternatively, an explanation of why a DMC is not needed | Page 11 (Quality control measurement) |
|  | 21b | Description of any interim analyses and stopping guidelines, including who will have access to these interim results and make the final decision to terminate the trial | N/A |
| Harms | 22 | Plans for collecting, assessing, reporting, and managing solicited and spontaneously reported adverse events and other unintended effects of trial interventions or trial conduct | N/A |
| Auditing | 23 | Frequency and procedures for auditing trial conduct, if any, and whether the process will be independent from investigators and the sponsor | Page 11 (Quality control measurement) |
| Ethics and dissemination | | |  |
| Research ethics approval | 24 | Plans for seeking research ethics committee/institutional review board (REC/IRB) approval | Page 11 (Ethical consideration) |
| Protocol amendments | 25 | Plans for communicating important protocol modifications (eg, changes to eligibility criteria, outcomes, analyses) to relevant parties (eg, investigators, REC/IRBs, trial participants, trial registries, journals, regulators) | N/A |
| Consent or assent | 26a | Who will obtain informed consent or assent from potential trial participants or authorised surrogates, and how (see Item 32) | Page 11 (Ethical consideration) |
|  | 26b | Additional consent provisions for collection and use of participant data and biological specimens in ancillary studies, if applicable | N/A |
| Confidentiality | 27 | How personal information about potential and enrolled participants will be collected, shared, and maintained in order to protect confidentiality before, during, and after the trial | Page 11 (Ethical consideration) |
| Declaration of interests | 28 | Financial and other competing interests for principal investigators for the overall trial and each study site | Page 21 (Competing interests) |
| Access to data | 29 | Statement of who will have access to the final trial dataset, and disclosure of contractual agreements that limit such access for investigators | Page 10 (Data management plan) |
| Ancillary and post-trial care | 30 | Provisions, if any, for ancillary and post-trial care, and for compensation to those who suffer harm from trial participation | N/A |
| Dissemination policy | 31a | Plans for investigators and sponsor to communicate trial results to participants, healthcare professionals, the public, and other relevant groups (eg, via publication, reporting in results databases, or other data sharing arrangements), including any publication restrictions | Page 12 (Reporting of the result) |
|  | 31b | Authorship eligibility guidelines and any intended use of professional writers | No professional writers will be used. |
|  | 31c | Plans, if any, for granting public access to the full protocol, participant-level dataset, and statistical code | Page 10 (Data management plan) |
| Appendices |  |  |  |
| Informed consent materials | 32 | Model consent form and other related documentation given to participants and authorised surrogates | Will be provided on request |
| Biological specimens | 33 | Plans for collection, laboratory evaluation, and storage of biological specimens for genetic or molecular analysis in the current trial and for future use in ancillary studies, if applicable | N/A |

*It is strongly recommended that this checklist be read in conjunction with the SPIRIT 2013 Explanation & Elaboration for important clarification on the items. Amendments to the protocol should be tracked and dated. The SPIRIT checklist is copyrighted by the SPIRIT Group under the Creative Commons “[Attribution-NonCommercial-NoDerivs 3.0 Unported](http://www.creativecommons.org/licenses/by-nc-nd/3.0/)” license.
